# Pearls & Oy-sters: Increased Visibility of Focal Cortical Dysplasia in Cerebral MRI During the First Year of Life

**DOI:** 10.1212/WNL.0000000000210128

**Published:** 2024-12-27

**Authors:** Hishayine Visvaratnam, Christian M. Korff, Margitta Seeck, Aikaterini Fitsiori

**Affiliations:** 1 Radiology Unit, Diagnostic Department, University Hospitals of Geneva, Switzerland;; 2 Faculty of Medicine, University of Geneva, Switzerland;; 3 Neuropediatrics Unit, Woman, Child and Adolescent Department, University Hospitals of Geneva, Switzerland;; 4 EEG and Epilepsy Unit, Department of Clinical Neurosciences, University Hospitals of Geneva, Switzerland; and; 5 Diagnostic and Interventional Neuroradiology Unit, Diagnostic Department, University Hospitals of Geneva, Switzerland.

## Abstract

Early detection of focal cortical dysplasia (FCD) using brain MRI in young children presenting with drug-resistant epilepsy may facilitate prompt surgical treatment, resulting in better control of seizures and decreased associated cognitive difficulties. Characteristics of FCD described in the literature are predominantly based on MRI findings in a fully myelinated brain; therefore, changes occurring during early brain maturation are not well known. In this case report, we describe distinct MRI features of a FCD visualized best before completion of myelination of the cortex and subcortical white matter. Specifically, this lesion exhibited increased visibility because of better delineation of the borders of the pathological area involved in the malformation compared with imaging obtained after completion of myelination. Although the pathophysiology of these imaging features necessitates further investigation, one hypothesis is that excessive neuronal input in young epileptic patients could trigger premature myelination of overstimulated fibers underlying the cortical epileptogenic focus. The aim of this case report was to raise awareness that seizures might induce local changes in brain myelination, which can be detected by MRI during the first year of life. Early identification of FCD may prompt surgical treatment in selected patients with drug-resistant epilepsy.

## Pearls

- Early detection of focal cortical dysplasia (FCD) in young children presenting with drug-resistant epilepsy may facilitate prompt surgical treatment, resulting in better outcomes.
- MRI plays an essential role in the diagnosis of FCD.
- Increased visibility of MRI signal abnormalities of the white matter (WM) adjacent to FCD can be detected on MRI performed during the first year of life.

## Oy-sters

- Characteristics of FCD described in the literature are predominantly based on MRI findings in a fully myelinated brain; therefore, changes occurring during brain maturation are not well known.

## Case Report

**Figure 1:**
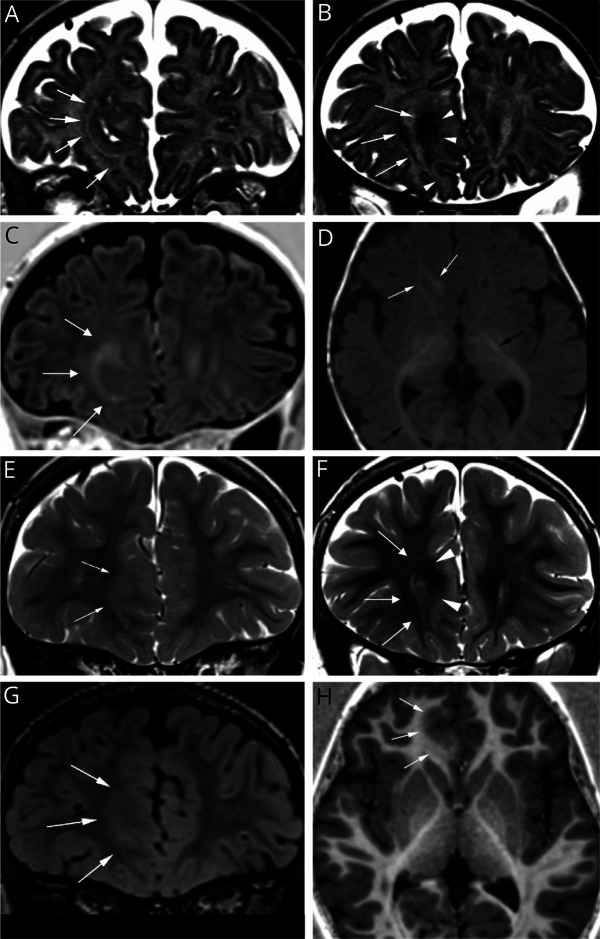
MRI Studies at the Age of 6 Months and 4 Years in a Girl With Refractory Epilepsy Related to Right Anterior Cingulate FCD Coronal T2 (A and B), coronal fluid attenuated inversion recovery (FLAIR) (C), and axial T1 (D) 1.5T brain MRI performed at 6 months of age showing an arciform (“comma shape”) T2 hypointensity in the WM underlying the right anterior cingulate gyrus (white arrows in A and B), missed during initial interpretation. These early changes seemed to better delineate the borders of the malformation extending to the right basal ganglia medially and to the right gyrus rectus caudally (white arrowheads in B) and corresponded well to the pathologic zone of the histologic specimen after surgical excision. Note the lesion has the same signal (white arrows in D) as the internal capsule and optic radiation (black arrows in D), which are normally myelinated at this age suggesting accelerated myelination in the FCD. No cortical thickening or signal abnormality of the cortex was observed on the initial MRI. Coronal T2 (E and F), coronal FLAIR (G), and axial T1 (H) 3T brain MRI scans performed with a specific epilepsy protocol at 4 years of age showing similar changes with high T2/FLAIR and relatively low T1 signal in the same area (white arrows). Note a cortical thickening of the right anterior cingulate gyrus, associated with subtle blurring of the gray-white matter junction (G and H, white arrows), an imaging hallmark of FCD. FCD = focal cortical dysplasia; WM = white matter.

**Figure 2:**
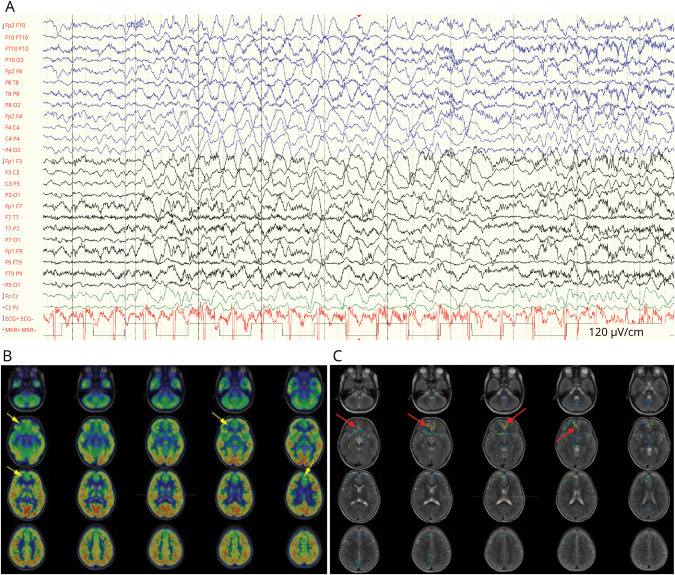
EEG and PET/CT Images in a Girl With Refractory Epilepsy Related to Right Anterior Cingulate FCD Ictal EEG (A) showing increased right frontotemporal activity (gain 120 μV/s with filters 4.0–200.0 Hz). Axial interictal fluorodeoxyglucose (FDG) PET-CT (B) and PET-CT/MRI fusion reconstruction (C) also consistent with an epileptic focus in the right frontomesial region (arrows).

A 5-month-old, developmentally appropriate girl was referred to our hospital after 1 week of multiple clusters of seizures. Events were increasing in frequency, initially 4 episodes/day escalating to 3–4 episodes/hour upon arrival. The seizures were characterized by hypertonic flexion of all limbs and facial erythema lasting 30 seconds to 1 minute without loss of consciousness, followed by postictal fatigue. Her personal and family history were otherwise unremarkable. Neurologic interictal examination and psychomotor development were normal. EEG on arrival revealed increased right frontotemporal activity, whereas interictal EEG was normal. Brain MRI scan (1.5T) performed at 6 months, interpreted by a pediatric radiologist, was initially reported as normal (Figure 1, A–D). Antiseizure drugs (carbamazepine first, followed by levetiracetam) were introduced, resulting in a significant decrease in seizure frequency, with only rare events (2–3/year) occurring mostly during viral infections. At the age of 4 years, a gradual recurrence of seizures with clusters of predominantly nocturnal seizures (up to 10/night) prompted adjustment of treatment, as well as phase 1 investigations including a 3T MRI with a specific epilepsy protocol. This second MRI demonstrated thickening of the right anterior cingulate cortex associated with subtle blurring of the gray-white matter (G/WM) junction (Figure 1, E–H), suggesting a diagnosis of right mesial frontal FCD. Of interest, retrospective analysis of the first MRI performed at the age of 6 months showed prominent focal changes in the subcortical WM of the area corresponding to the FCD, suggesting the diagnosis. These imaging findings were consistent with the patient's video-scalp EEG (Figure 2A) and interictal positron emission tomography-computed tomography (PET-CT, Figure 2B and C, eFigure 1) findings. Genetic analyses performed at 4 years were unrevealing. In view of the patient's refractory epilepsy and the excellent concordance of all assessments (eFigure 2), the multidisciplinary epilepsy team proposed surgical treatment, waiving a phase II investigation. Right anterior frontal cortical resection was performed at 6 years, and histologic examination confirmed a FCD type IIa, according to the International League Against Epilepsy classification.^1^ During the first 4 months after surgery, the patient remained seizure free with carbamazepine monotherapy, with no neurologic deficit or neurocognitive regression. However, high-frequency seizure recurrence (up to 3/day and 20/night) triggered hospital admission for treatment adjustment leading to a combination of carbamazepine, levetiracetam, and clobazam. Postsurgical MRI and PET-CT images demonstrated a small residual FCD lesion in the frontobasal region, consistent with the postsurgical recorded EEG focus. Six months after the first surgery, a second-look operation was performed, resulting in resection of the residual focus of FCD, confirmed by the histologic examination. In the immediate postoperative period, the patient was neurologically intact and seizure free under the preoperative triple antiepileptic treatment.

## Discussion

FCD corresponds to a malformation of cortical development characterized by disorganized cortical layers, dysmorphic neurons, and WM abnormalities.^2^ It is one of the most common causes of epilepsy eligible for surgical treatment in pediatric patients.^1^ Based on emerging knowledge of FCD's variable clinical, histologic, and radiologic manifestations, new consensus classifications were released in 2022.^1^ FCD type II is associated with better prognosis after surgery compared with FCD type I because of more accurate detection of the epileptic zone, with up to 91% of patients with FCD IIb being seizure free vs 68% of patients with FCD type IIa.^3^

Imaging characteristics of FCD described in the literature are based on MRI findings in a fully myelinated brain; therefore, changes occurring during brain maturation are not well known. The classical findings of FCD in the mature brain include cortical thickening, blurring of the G/WM junction, high T2 with respect to low T1 signal of the cortex and subcortical WM, as well as the presence of abnormal gyration or “transmantle sign,” which is a funnel-shaped T2/fluid attenuated inversion recovery (FLAIR) hyperintensity corresponding to the pathway of migration of the abnormal neurons from the ventricle toward the crown of a gyrus or bottom of a sulcus in certain cases.^4^ These classical findings can be overlooked in approximately 40% of patients with FCD type I and 10% of those with FCD type II,^5^ resulting in false-negative MRI scans. To increase their detection, the use of epilepsy-specific MRI protocols on high-field machines (optimally 3T or even 7T when available) is recommended, as well as interpretation of the scans by experienced neuroradiologists.^1^ Various technological tools based in deep learning with morphometric voxel-based analysis of the cortex and the G/WM junction are already in use in specialized centers.^6^ New advances in artificial intelligence seem promising for subtle FCD detection.^7^

This case exhibited distinct MRI features of FCD preceding the completion of myelination of the cortex and subcortical WM, with increased visibility compared with the classically described appearance of FCD after completion of myelination. The importance of this finding is that the extent of the area involved in the malformation was better demarcated in the early MRI performed at 6 months of age compared with the MRI performed at the age of 4 years. In the latter scan, the abnormalities became obscured by the myelination such that the only indication of the FCD was the blurring of the G/WM junction with difficult to identify borders. On the contrary, the arciform T2 hypointensity seen in the early images seemed to better delineate the borders of the malformation in a “comma shape” appearance (Figure 1), which corresponded to the pathologic zone of the histologic specimen after surgical excision. These early changes were subtle and could be easily missed without relevant clinical information on the seizures' focus, underlining the importance of close collaboration between radiologists and neurologists in suspected focal epilepsy cases.

The mechanism behind the signal abnormalities seen at 6 months is unknown. Based on the fact that the MRI signal of WM undergoes changes from hyperintense to hypointense in T2 and from hypointense to hyperintense in T1 weighting during myelination,^8^ we speculate that the T2 and T1 shortening seen in the initial MRI in this case might suggest an accelerated myelination within the WM area underlying the cortical development malformation compared with the surrounding normal WM. An argument to support this hypothesis is that the intensity of the pathologic area is the same signal in T1 and T2 images as brain structures normally myelinated at this age, such as the anterior and posterior limb of the internal capsule and the optic radiation^8^ (Figure 1D). This hypothesis was suggested in 1998 by some authors, who proposed that the MRI signal abnormalities of WM underlying FCD in a two-month-old boy with refractory epilepsy were secondary to seizure-induced premature myelination of overstimulated fibers.^9^ Similar findings were described in a small case series, although the authors note that the signal abnormalities could be inherent features of the dysplastic brain rather than transient seizure-induced changes.^10^ Notably, general hypermyelination or advanced myelination have also been associated with Sturge-Weber disease^11^ and hemimegaloencephaly,^12^ conditions which usually manifest with a high frequency of daily seizures. Finally, some preclinical studies in mice have demonstrated that absence-type of seizures can induce activity-dependent myelination within the seizure network.^13,14^

Alternative explanations for signal changes in FCD including blood extravasation, protein deposition, and dystrophic mineralization, are contested and seem less probable.^9^ The hypothesis of extravascular blood deposition seems unlikely because of the absence of typical T1 and T2 signal changes after hemoglobin's degradation. The hypothesis of dystrophic mineralization seems also unlikely because there was no evidence of calcification in the CT and MRI scans, nor in the histologic analysis of the specimen. Protein deposition could not be excluded, as a transient phenomenon explaining the disappearing changes in later MRI; some focal volume reduction is usually associated, though, with this abnormality,^9^ not seen in our case in the follow-up MRI.

Myelination of the brain mirrors its functional maturity. However, the entirety of imaging changes during this process is still not completely understood. Our case report describes FCD best seen with MRI performed within the first year of life. Appreciation of these changes could not only allow for an early diagnosis of FCD but also for a better delineation of the abnormal brain parenchyma involved in the malformation when surgical resection is envisioned, leading to optimal seizure control. The diagnosis of a focal lesion on MRI typically prompts a complete presurgical evaluation in children with refractory seizures. A potentially curative resection can be envisaged in a significant number of such patients, at any age including during their first year of life. Surgical procedures are no longer considered as last resort approaches in such situations and are recommended to be performed as early as possible to avoid the harmful consequences of incomplete control of epileptic activity. This case report aims at raising awareness that seizures can induce local changes in brain myelination and that these changes can be detected by MRI during the first year of life. Changing our approach to early and full imaging workup in selected cases of severe forms of infantile epilepsy^15^ may improve the outcome of this otherwise deleterious disease, both on a neurologic and cognitive level.

## Informed Consent for Participation in Research Studies

The authors state that they obtained the written informed consent of the patient as well as of the patient's legally authorized representative for publication of this report and that this study is exempt from ethics board review approval (Commission Cantonale d'Ethique de la Recherche sur l'être humain).
